# CONSTIPATION SCORING SYSTEM VALIDATED FOR THE PORTUGUESE LANGUAGE (*ÍNDICE DE GRAVIDADE DA CONSTIPAÇÃO INTESTINAL*): IS IT RELIABLE IN ASSESSING THE SEVERITY OF INTESTINAL CHRONIC CONSTIPATION IN OUR POPULATION?

**DOI:** 10.1590/0102-672020230067e1785

**Published:** 2024-03-15

**Authors:** Ilario FROEHNER, José Marcio Neves JORGE, Carlos Frederico Sparapan MARQUES, Vera Lúcia Conceição de Gouveia SANTOS, José JUKEMURA

**Affiliations:** 1 Universidade de São Paulo, Faculty of Medicine, Department of Gastroenterology - São Paulo (SP), Brazil;; 2 Universidade de São Paulo, School of Nursing, Medical-Surgical Nursing Department - São Paulo (SP), Brazil.

**Keywords:** Constipation, Validation Study, Indicators of Quality of Life, Severity of Illness Index, Constipação Intestinal, Estudo de Validação, Indicadores de Qualidade de Vida, Índice de Gravidade de Doença

## Abstract

**BACKGROUND:**

There is a lack of valid and specific tools to measure chronic constipation severity in Brazil.

**AIMS:**

To validate the Constipation Scoring System for Brazilian spoken Portuguese.

**METHODS:**

Translation, cultural adaptation, and validation itself (reliability and convergent and divergent validation). Translation: definitive version from the original version’s translation and evaluation by specialists. Cultural adaptation: score content analysis of the definitive version, as an interview to patients. Interobserver reliability: application by two researchers on the same day. Intraobserver reliability: same researcher at different times, in a 7-day interval. Divergent validation: non-constipated volunteers. Convergent validation: two groups, good response to clinical treatment and refractory to treatment.

**RESULTS:**

Cultural adaptation: 81 patients, 89% female, with mean age of 55 and seven years of schooling, and overall content validity index was 96.5%. Inter and intraobserver reliability analysis: 60 patients, 86.7% female, mean age of 56 and six years of schooling, and the respective intraclass correlation coefficients were 0.991 and 0.987, p<0.001. Divergent validation: 40 volunteers, 25 male, mean age of 49 years, and the mean global score was 2. Convergent validation of patients with good response to clinical treatment: 47 patients, 39 female, mean age of 60 and six years of schooling, and the pre- and post-treatment scores were 19 and 8, respectively (p<0.001). Convergent validation of refractory to clinical treatment patients: 75 patients, 70 female, mean age of 53 and seven years of schooling, and the global average score was 22.

**CONCLUSIONS:**

The Constipation Scoring System (Índice de Gravidade da Constipação Intestinal) validated for the Brazilian population is a reliable instrument for measuring the severity of intestinal chronic constipation.

## RESUMO

**RACIONAL:**

No Brasil há escassez de instrumentos específicos e validados para a avaliação da gravidade da constipação intestinal crônica.

**OBJETIVOS:**

Validar o instrumento *Constipation Scoring System* para pacientes com constipação crônica.

**MÉTODOS:**

Tradução, adaptação cultural e validação propriamente dita. Tradução: versão definitiva a partir de traduções do original avaliadas por especialistas. Adaptação cultural: avaliação do conteúdo por entrevista a pacientes. Confiabilidade interobservadores: entrevista por dois pesquisadores no mesmo dia. Confiabilidade intraobservador: duas entrevistas pelo mesmo pesquisador (intervalo de 7 dias). Validação divergente: voluntários não constipados. Validação convergente: dois grupos, boa resposta e refratários ao tratamento clínico.

**RESULTADOS:**

Adaptação cultural: 81 pacientes, sendo 89% do sexo feminino, com média de idade de 55 anos e 7 anos de escolaridade. O índice de validade de conteúdo global foi de 96,5%. Confiabilidade interobservadores e intraobservador: 60 pacientes, sendo 86,7% do sexo feminino, com média de idade de 56 anos e 6 anos de escolaridade. O coeficiente de correlação intraclasse foi de 0,991 e 0,987 (p<0,001), respectivamente. Validação divergente: 40 voluntários, sendo 62,5% do sexo masculino, com média de idade de 49 anos e pontuação média: 0. Validação convergente dos pacientes com boa resposta do tratamento clínico: 47 pacientes, sendo 83% do sexo feminino, com média de idade de 60 anos e 6 anos de escolaridade. Os índices pré e pós-tratamento foram 19 e 8 (p<0,001), respectivamente. Validação convergente dos pacientes refratários ao tratamento clínico: 75 pacientes sendo 93% do sexo feminino, com média de idade de 53 anos e 7 anos escolaridade. A pontuação média foi 22.

**CONCLUSÕES:**

O *Constipation Scoring System* validado para população brasileira (Índice de Gravidade da Constipação Intestinal), é instrumento confiável para a aferição da gravidade da constipação intestinal crônica.

## INTRODUCTION

Intestinal chronic constipation is one of the most frequent gastrointestinal disorders in general clinical practice^17^ ^,^ ^27^ ^,^ ^28^, reaching up to 34% of the studied population. It is considered to have a high global prevalence being endemic in the elderly population and associate with a significant impact on quality of life^14^ ^,^ ^16^.

It basically consists of intestinal evacuation disorders, either due to low frequency of bowel movements - slow transit constipation, or because of difficulty in expelling the fecal bolus - obstructed defecation, or the combination of both, called mixed^4^.

A great diversity of definitions and a consequent difficulty in comparing results culminated in Rome’s diagnostic criteria, which are evacuation effort, hard stools, feeling of incomplete or obstructed defecation, need for manual maneuvers to aid evacuation and less than three bowel movements per week^9^ ^,^ ^10^.

Rome criteria help in the diagnosis of intestinal chronic constipation; however, they do not grade or stratify its clinical severity^8^ ^,^ ^29^. For this purpose, from the 1980s onwards, the first severity scores were created. Most severity scores with regard to intestinal chronic constipation were created for specific populations, for example, that suffering from Parkinson’s disease^6^.

Currently, in Brazil, there is a shortage of specific and validated instruments for the assessment of functional constipation.

The Constipation Scoring System was proposed in 1996 by Agachan et al.^1^, Cleveland Clinic Florida researchers, as a tool for measuring functional intestinal chronic constipation, without being directed towards any specific group. The enrolled patients had their diagnosis through complementary exams, given the fact that the Rome I diagnostic criteria were recently published in 1994.

Formed by only eight questions and with an accuracy of 96%, it gained popularity among research centers and health professionals worldwide. The metrics evaluated are frequency of bowel movements, painful evacuation effort, feeling incomplete evacuation, abdominal pain, minutes in lavatory per attempt, type of assistance to defecate (manual, laxatives, or enemas), unsuccessful attempts for evacuation per 24 hours and duration of constipation (Table 1).

**Table 1 -:** Constipation Scoring System (Minimum Score 0; Maximum Score 30)^1^.

	Score
Frequency of bowel movements	
1-2 times per 1-2 days	0
2 times per week	1
Once per week	2
Less than once per week	3
Less than once per month	4
Difficulty: painful evacuation effort	
Never	0
Rarely	1
Sometimes	2
Usually	3
Always	4
Completeness: feeling incomplete evacuation	
Never	0
Rarely	1
Sometimes	2
Usually	3
Always	4
Pain: abdominal pain	
Never	0
Rarely	1
Sometimes	2
Usually	3
Always	4
Time: minutes in lavatory per attempt	
Less than 5	0
5-10	1
10-20	2
20-30	3
More than 30	4
Assistance: type of assistance	
Without assistance	0
Stimulative laxatives	1
Digital assistance or enema	2
Failure: unsuccessful attempts for evacuation per 24 hours	
Never	0
1-3	1
3-6	2
6-9	3
More than 9	4
History: duration of constipation (years)	
0	0
1-5	1
5-10	2
10-20	3
More than 20	4
Total score	

Each metric has a minimum and a maximum score, from 0 to 4 or 4, respectively. The sum of the score of each item, if greater than 15, out of a total value of 30, is diagnostic of intestinal chronic constipation and stratifies its severity.

Cultural adaptation and validation of an instrument allows the use of the same tool in different cultural contexts, avoids the creation of countless other questionnaires, favors the standardization of information, and permits data comparison and multicenter studies^12^ ^,^ ^15^.

The objective was to validate the Constipation Scoring System for assessing the severity of intestinal chronic constipation in patients in the Brazilian environment, context, and culture.

## METHODS

This research was approved by the Ethics Committee for the Analysis of Research Projects of the University Hospital of the Universidade de São Paulo - CAPPesq/HC-FMUSP (Service Instruction number 01/98, and numbers 1,864,046 and 61097912.0.0000.0068).

Participants were volunteers and intestinal chronic constipated patients. Throughout this study, all information obtained was acquired by applying the questionnaire as an interview from March 2012 to February 2013.

Volunteer inclusion criteria:

- Individuals aged 18 years or over, employed at Hospital das Clínicas of the Universidade de São Paulo or patients’ companions treated in it.
- Necessarily, the diagnosis of intestinal chronic constipation according to Rome III criteria^9^ ^,^ ^10^ was not fulfilled, namely: onset of symptoms at least six months earlier and lasting at least three months, and two of the following criteria: less than three bowel movements per week, manual assistance to ease defecation, straining, hard stools, and feeling of incomplete or obstructed evacuation in 25% or more of the bowel movements.

Volunteer exclusion criteria:

- Individuals with cognitive alterations that prevent understanding the questions posed by the interviewer and their answers.
- Non-agreement with the Informed Consent Form content.

Patient inclusion criteria:

- Individuals aged 18 years or over, diagnosed with intestinal chronic constipation according to Rome III criteria^9^ ^,^ ^10^, as described above.

All of them under medical care at the Colon, Rectum and Anus Physiology Ambulatory of the Coloproctology Division at the Faculty of Medicine of the Universidade de São Paulo.

- Consecutively enrolled in the present study according to the Ambulatory (cited above) routine.

Patient exclusion criteria:

- Individuals with cognitive alterations that prevent adequate understanding of the questions posed by the interviewer and their answers.
- Non-agreement with the Informed Consent Form content.

A good response to clinical treatment was considered when the application of the Rome III criteria^9^ ^,^ ^10^ no longer indicated the diagnosis of intestinal chronic constipation for at least six months of follow-up, in a therapeutic maintenance regimen.

Patients refractory to clinical treatment were those who still had the diagnosis, according to Rome III criteria^9^ ^,^ ^10^, four weeks after starting treatment.

The validation of the Constipation Scoring System followed this respective sequence: translation and reverse translation, cultural adaptation, and validation itself, which includes the assessment of intra and interobserver reliability, divergent validation, convergent validation of patients with good response to clinical treatment and convergent validation of refractory to clinical treatment patients^3^ ^,^ ^15^.

The original score was translated into Portuguese in two versions, one by a coloproctologist fluent in English and experienced in anorectal physiology, and another by a public translator.

The two available versions, together with the original score, were evaluated and compared by three coloproctologists experienced in anorectal physiology and by a nurse specializing in intestinal stomas, urinary and anal continence. Each evaluator stated their modifications, which were compiled in a new document, with the addition of a proposal for a new evaluation by the team and the subsequent creation of a consensus version.

Two translations into English of the consensus version were performed by two English teachers, without any information about the study and the original score^15^.

The original and all other versions were compared to determine the grammatical and semantic equivalence between them. After team agreement, the final version in Portuguese was established.

Cultural adaptation verified the final version’s content validity, the content validity index (CVI), in which each item of the questionnaire must adequately express the variable to be measured associated with proper understanding by the interviewee. Values above 80%^15^ were accepted.

Cultural adaptation occurred by the application of the final version by the researcher, in the form of an interview. The option “I did not understand” was added to each of the questionnaire items, marked by the researcher when the patient found it difficult to understand an item. If 15% or more of the patients did not understand the same question (“I did not understand” option marked), it would be reviewed by the team and reapplied to them.

Validation determines the ability of the research instrument to measure what it was proposed for. Validation itself consists of evaluating the reliability or reproducibility, the divergent validity and convergent validity of patients with good response to clinical treatment and convergent validity of patients refractory to clinical treatment.

Reliability is the ability of a survey instrument to measure similar data over time regardless of the interviewer. It was evaluated by applying the questionnaire to new patients in three moments, called “Measurement 1, 2 and 3”.

At first, they responded to the researcher (Measure 1-M1). Immediately afterwards, in another room and without the researcher’s knowledge, to one of the coloproctologists (Measurement 2-M2). In the third moment, after one week, the questionnaire was again applied to the patients by the researcher (Measure 3-M3).

M1 versus M2 analysis referred to the interobserver assessment, verifying adequate reproducibility regardless of the interviewer. M1 versus M3 analysis referred to the intraobserver assessment, which verifies the tool reproducibility over time.

Reliability in both analyses was considered adequate if the intraclass correlation coefficient (ICC) in both was greater than 75%, indicating high reproducibility.

Divergent validity tests the relationship between the instrument and the opposite or absent clinical picture. In this scenario, the questionnaire is valid when it presents significantly reduced scores. In the absence of intestinal constipation, the score must present values lower than 15 points^1^. The final version was applied by the researcher in the form of an interview to volunteers.

Convergent validity tests the questionnaire with another one aimed at the same purpose or with obvious situations, as in this study, with patients who had a good response to clinical treatment and with patients refractory to clinical treatment.

In those with good clinical response, the valid research instrument should indicate the improvement of the clinical picture through the significant decrease in its score.

The final version was applied by the researcher, in the form of an interview, to patients with good clinical response. The initial interview during the first appointment was called “pre-treatment” (T1) and, during the follow-up, “post-treatment” (T2).

In patients refractory to the clinical treatment, the research instrument must present a final score still maintaining the diagnosis of intestinal chronic constipation and its severity. The greatest severity was considered in those patients with an initial assessment greater than 20 points. The final version was applied by the researcher, in the form of an interview, to patients refractory to clinical treatment.

The data analysis process started with a descriptive exploration, including absolute and relative frequency for qualitative attributes. The summary of discrete or continuous quantitative measures was performed with mean, standard deviation (SD), median, 25^th^ percentile and 75^th^ percentile.

The eight questions were characterized as “Q1” for question 1 and successively up to “Q8” for question 8.

In the cultural adaptation stage, the CVI was applied. This index measures the proportion of participants who agree on certain aspects of the instrument and its items; the statistic that initially allows analyzing each item individually and then the instrument as a unity. This method employs a Likert-type scale with scores from one to four, which is the highest intensity or quantity. In this study, questions with CVI <0.8 or 80% were reviewed and reassessed.

The Bland-Altman approach was used to represent the agreement between Likert scale scores for the test-retest (intraobserver) and interobserver assessment. Bland-Altman scatter plots were used to exemplify the analytical process in two different examples for each of the approaches (intraobserver and interobserver).

The two-way, mixed-effects, single measure intraclass correlation coefficient (ICC) and respective 95% confidence intervals (95%CI) were used to analyze intraobserver and interobserver reliability. The classification indicated for the ICC interpretation was the following: ICC lower than 0.40 indicates low reproducibility; ICC between 0.40 and 0.75 indicates moderate reproducibility; ICC >0.75 indicates high reproducibility.

To assess the hypothesis of equality between pre- and post-treatment times in the convergent validation of patients with good response to clinical treatment, as well as in the evaluation of the absence of the condition (divergent validation), the paired Wilcoxon test was used. All tests carried out considered a bidirectional a of 0.05 and a 95% confidence interval and were performed with computational support from IBM Statistical Package for the Social Sciences - SPSS^®^ 25 and Excel 2016^®^ (Microsoft Office).

In the design of the present study, the determination of sample calculations for the researched groups was not carried out due to its purpose: agreement, similarity and compatibility between instrument measurements and not obtaining incidence, prevalence, or risks. Even so, the number of participants in all stages of the study was greater than 30, a sample considered significant in instrument validation studies^5^.

## RESULTS

The first English-Portuguese translation was the one used by the Colon, Rectum and Anus Physiology Ambulatory of the Coloproctology Division, at the Faculty of Medicine of the Universidade de São Paulo, considered the reference version so far.

The Table 2 describes the final version of the Constipation Scoring System^1^ translated into Portuguese spoken in Brazil, the *Índice de Gravidade da Constipação Intestinal*.

**Table 2 -:** Final version of the Constipation Scoryng System’s translation to Brazilian spoken Portuguese - *Índice de Gravidade da Constipação Intestinal* (Minimum Score 0; Maximum Score 30).

Frequência das evacuações	Pontuação
1 ou 2 vezes a cada 1 a 2 dias	0
2 vezes por semana	1
1 vez por semana	2
Menos de 1 vez por semana	3
Menos de 1 vez por mês	4
Esforço evacuatório desconfortável ou doloroso	Pontuação
Nunca	0
Raramente	1
Às vezes	2
Geralmente	3
Sempre	4
Sensação de evacuação incompleta	Pontuação
Nunca	0
Raramente	1
Às vezes	2
Geralmente	3
Sempre	4
Dor abdominal ao evacuar	Pontuação
Nunca	0
Raramente	1
Às vezes	2
Geralmente	3
Sempre	4
Tempo no banheiro para evacuar (em minutos)	Pontuação
Menos de 5 minutos	0
De 5 a 10 minutos	1
De 10 a 20 minutos	2
De 20 a 30 minutos	3
Mais de 30 minutos	4
Uso de laxantes, enemas ou ajuda com as mãos	Pontuação
Sem ajuda	0
Uso de laxantes	1
Uso de enemas ou ajuda com as mãos	2
Tentativas malsucedidas (fracassadas) para evacuar (vezes por dia)	Pontuação
Nunca	0
1 a 3	1
3 a 6	2
6 a 9	3
Mais de 6	4
Duração da constipação (em anos)	Pontuação
Zero	0
1 a 5	1
5 a 10	2
10 a 20	3
Mais de 20	4
Total	

A total of 303 individuals, 263 patients and 40 volunteers were interviewed.

**Figure 1 -:**
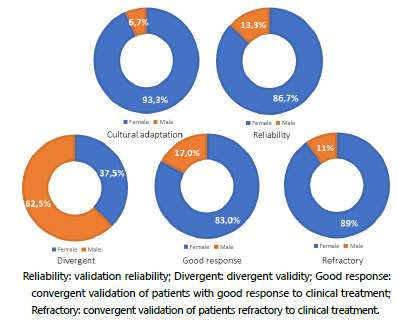
Gender distribution ratio according to study groups. Groups involving patients presented a bigger proportion of females, conversely to volunteers’ group (divergent).

**Figure 2 -:**
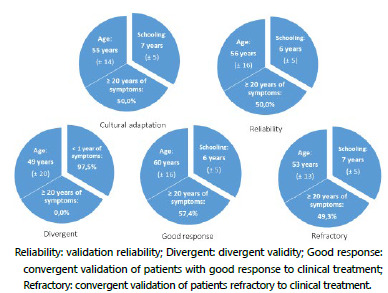
Study groups demographics. There is a homogeneity concerning age, symptoms duration and schooling among patients (Cultural adaptation, Reliability, Good Response and Refractory). In “Divergent”, volunteers presented symptoms for less than one year.

In the cultural adaptation, 81 patients were included, 73 (89%) females and nine (11%) males. Mean age was 55 years (±14 years) and schooling was seven years (±5 years). The duration of symptoms in these patients was heterogeneous, with 41 patients having equal or more than 20 years of symptoms (Figures 1 and 2).

Global content validity index was 96.5% (Table 3). In questions Q3 and Q7, respondents reported less understanding, 87.8% (IC95% 79.4-93.6%) and 86.6% (IC95% 78-92.7%), respectively.

**Table 3 -:** Content validity index to patients’ question comprehension - total and per question data.

CVI*	n	%	95%CI	
			Inferior (%)	Superior (%)
Q1	82	100.0	-	-
Q2	81	98.8	94.4	99.9
Q3	72	87.8	79.4	93.6
Q4	82	100.0	-	-
Q5	82	100.0	-	-
Q6	82	100.0	-	-
Q7	71	86.6	78.0	92.7
Q8	81	98.8	94.4	99.9
IVC^†^	633	96.5	95.3	97.8

*Content validity index per question; ^†^ Global content validity index. CVI: Content validity index; n: number of patients; 95%CI: 95% confidence interval; Q1-Q8: question 1 to question 8, respectively.

In reliability - interobserver and intraobserver analysis -, 60 patients were interviewed, 52 (86.7%) females and eight (13.3%) males, with a mean age of 56 years (±16 years), six years of schooling (±5 years), and half of them had had symptoms for 20 years or longer (Figures 1 and 2).

Mean score on measure 1 (M1) was 20 (±5), on measure 2 (M2) it was 20 (±5) and on measure 3 (M3) it was 20 (±5).

**Figure 3 -:**
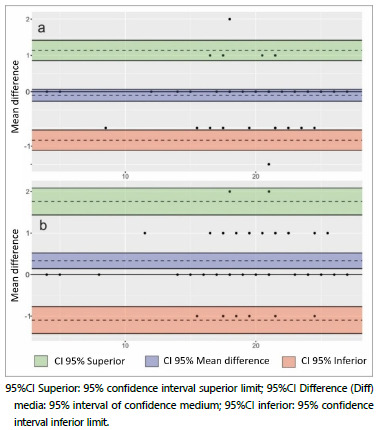
Validity, Reliability - interobserver (a) and intraobserver (b) analysis according to Bland-Altman test. The Bland-Altman analysis for the interobserver measurements showed only two discordant points beyond the graphic acceptance regions (a). In the case of the intraobserver analysis, no point was observed beyond the areas of graphic acceptance (b).

The Bland-Altman analysis for the interobserver measurements showed only two discordant points beyond the graphic acceptance region - between 95%CI superior border and 95%CI inferior border (Figure 3a). In the case of the intraobserver analysis, no point was observed beyond this area (Figure 3b).

Overall ICC for the interobserver analysis was 0.991 (p<0.001) and 0.987 (p<0.001) for the intraobserver analysis. In the interobserver analysis, the lowest ICC was 0.890 for Q7, while in the intraobserver analysis it was 0.774 for Q7 and 0.687 for Q6, both still statistically significant (p<0.001).

In the divergent validation, 40 volunteers were interviewed, 25 (62.5%) males and 15 (37.5%) females, with a mean age of 49 years (±20 years) and most of them, 39 (97.5%), with less than one year of symptoms (Figures 1 and 2).

**Figure 4 -:**
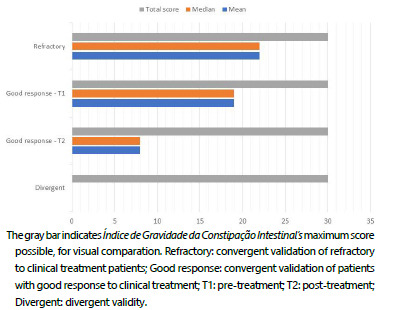
Study groups scoring. “Refractory” presented higher mean and median values. In “Good response”, between “T1” and “T2”, scoring decreased significantly due to the clinical treatment. “Divergent” presented zero score, because of the absence of intestinal chronic constipation in volunteers.

Mean and median scores for all questions in the subject pool were zero. Questions Q2, Q3, Q4, Q5, Q6 and Q8 had a maximum score of 2 points, while Q1 and Q7 had a maximum score of 1 point. The minimum and maximum values were 0 and 10 (Figure 4).

In the convergent validation of patients with good response to clinical treatment, 47 patients were interviewed, 39 (83%) females and eight (17%) males, with mean age of 60 years (±16 years), six years of schooling (±5 years), and almost 60% had symptoms duration equal to or longer than 20 years (Figures 1 and 2).

**Figure 5 -:**
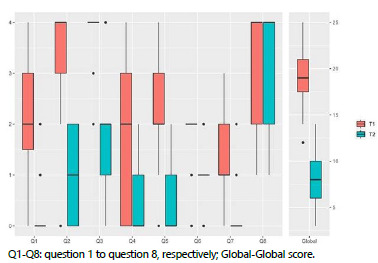
Global scores and per question scores - pre-treatment (T1) and post-treatment (T2) in convergent validation of patients with good response to clinical treatment.

**Figure 6 -:**
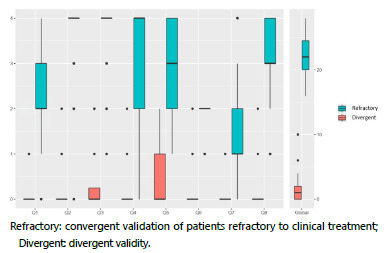
Global scores and per question scores - refractory validity (*Refratário*, in blue) and divergent validity (*Divergentes*, in pink). Q1-Q8: question 1 to question 8, respectively.

Pre-treatment global analysis (T1) showed a mean score of 19 (±3) and a median score of 19 (17-21), while post-treatment analysis (T2) showed a drop in score to a mean of 8 (±3) and a median of 8 (6-10), p<0.001. Furthermore, the analysis by question showed that Q8 was the only one that did not present a significant change after treatment, with a mean of 3 (±1) both in T1 and T2, p=0.988 (Figure 5). It is possible to verify the global pre- versus post-treatment comparison and the analysis by question in Figure 6.

In the convergent validation of refractory to clinical treatment patients, 75 individuals were interviewed, 70 (93.3%) females and five (6.7%) males, with a mean age of 53 years (±13 years), seven years of schooling (±5 years) and almost 50% with symptoms duration equal to or longer than 20 years (Figures 1 and 2).

The mean global score was 22 (±3), with a median of 22 and an interquartile range from 20 to 25 (Figure 4). Questions Q2 and Q3 resulted in a mean of 4 (±1) and both had a median and interquartile range of 4. Except for Q6, all other questions had a maximum score of 4 points.

Figure 4 shows the scores in the refractory group and the divergent validation group in parallel. In the global score, it is possible to verify the difference between the two groups, which includes the divergent group with a median close to zero and the refractory group with a median greater than 20 points.

## DISCUSSION

Intestinal chronic constipation, mainly idiopathic type, is related to a significant subjective aspect. Important authors and studies deal with the perception of the patient considering herself/himself constipated based on cultural and personal definitions^4^ ^,^ ^16^. It depends on the health professional to improve communication, so that it is possible and fruitful. For patients with reduced schooling, for instance, it is necessary to be careful with words, expressions and technical terms that may not be completely understood^15^.

The scores, both diagnostic ones, such as Rome^9^ ^,^ ^10^, and severity ones, such as the Constipation Scoring System^1^, play the role of making information perceptible, objective, and clear for professionals and patients.

There are instruments that do not serve their purpose properly when translated literally, either by word composition or by meaning. An important example occurred in the validation for the Portuguese language of the Fecal Incontinence Quality of Life questionnaire, whose item “2 g”, with the literal translation “it is important for me to plan what I am going to do according to my intestinal functioning” resulted in 60% of misunderstanding due to the more complex structure of the words. Item “3l” had 100% of misunderstanding due to the translation “I avoid traveling by train or plane”, since the interviewees did not commonly use trains or planes, a fact related to a socioeconomic and geographical aspect of the origin of the instrument^35^.

The Constipation Scoring System^1^ was published in 1996 and was preceded by two other tools for nurses. The first one had the purpose of diagnosing intestinal constipation^26^ and the second one, the Constipation Assessment Scale, was a tool for assessing the severity of intestinal constipation. Both were obtained from the most common symptoms according to a review of the current literature^25^.

As a severity score, the Constipation Scoring System emerged from a differentiated methodology, based on patients with intestinal chronic constipation with a diagnosis formed by complementary exams. It is worth mentioning that the Rome I criteria were published only two years before, in 1994, a time when there was still a significant miscellany of empirical diagnostic criteria based on the personal experiences of health professionals. The selection of patients only with the diagnosis confirmed by complementary tests excluded empirical biases and allowed greater control of symptoms severity. Since then, it has become one of the most used indices of constipation severity to date^1^.

Additionally, regarding intestinal chronic constipation, unlike what was validated in this study, most severity indices were created with specific objectives and needs, mostly for specific populations, such as for some neuropathies, for example^6^.

The current scarcity of instruments for clinical measurement of intestinal chronic constipation severity in our country, the need to use such a tool both in the scientific environment and in daily practice, and the existence of an instrument already established in the scientific and clinical field created from an adequate methodology with significant accuracy highlights the importance of the instrument’s adaptation to the Portuguese language and proper validation.

The translation of the Constipation Scoring System into Portuguese spoken in Brazil consisted of several steps to obtain the consensual version, that is, one that generated agreement among multidisciplinary specialists in the area. The objective was to avoid conceptual discrepancies and misunderstanding by patients, as described above.

When validating an instrument, the population sample to be analyzed is essential. In the present study, the mean education of patients ranged from six to seven years, indicating that most had incomplete primary education.

The Brazilian population presents a significant sociocultural and schooling diversity. The more comprehensible the tool, the more adapted to a particular population it is. If any instrument is adapted and validated for a sample with a low level of education, its applicability can be inferred for almost the entire local population^15^ ^,^ ^30^.

In the context of the validation of the Constipation Scoring System, the application of the questionnaire through interviews by specialists allowed a better interpretation of patients’ and volunteers’ responses. Cultural adaptation is a paramount stage for the continuity of the validation, since it represents the accuracy of the index when applied to the desired population.

In our study, carried out in a specialized anorectal physiology center, the proportion of women is higher, accentuating the trend of a higher female prevalence, from the rural population to the tertiary center. The evaluation of 2,846 individuals from the rural area of Pelotas, state of Rio Grande do Sul, found a prevalence of 26.9% (36.8% of women and 13.9% of men, female-male ratio: 2.5-1)^7^. In an Italian colorectal tertiary center, 76% of the patients with intestinal chronic constipation were female, a ratio female-male of 3,16-1, respectively^32^. Conversely, in the groups that involved patients, the vast majority of volunteers are male, reinforcing the lower prevalence of intestinal chronic constipation in this gender^4^ ^,^ ^16^.

Likewise, with regard to patients, a significant number informed a duration of symptoms greater than 20 years. This fact infers the greater reliability and precision in obtaining information and emphasizes the chronic nature of intestinal constipation^1^ ^,^ ^4^ ^,^ ^16^ ^,^ ^23^ ^,^ ^28^.

Additionally, comparing the demographic data of all groups of patients, an expressive homogeneity was noticed in gender, age group, education, and symptoms duration, denoting sample reliability.

Considering the Score’s translation, this consensus version had as its main objective to adjust to the study population, aiming at a broad national reach, and maintaining the scientific clarity of the information. The title and the eight questions that form this measurement instrument were considered “concepts”, whose idea needed to be conveyed objectively and to have an unequivocal interpretation by the interviewee^3^ ^,^ ^15^.

For the consensus version, the Portuguese title chosen was “*Índice de Gravidade da Constipação Intestinal*”, a self-explanatory name, avoiding anglicisms and mentioning the aspect of evaluation the intestinal constipation severity and not just its diagnosis. The term “*escore*” was avoided, being properly replaced by “*índice*”, with the intention of indicating the severity of intestinal constipation. The word “*gravidade*”, which does not appear in the original title in English, is traditionally used by the Colon, Rectum and Anus Physiology Ambulatory of the Coloproctology Division at the Faculty of Medicine of the Universidade de São Paulo, and was accepted to reinforce the tool’s usefulness in grading the intensity of constipation, not diagnosing it. The word “system”, or “*sistema*” in Portuguese, had its purpose sufficiently fulfilled by the term “*índice*”. The word “*intestinal*” was added to clarify the use for the digestive system, avoiding any association with respiratory symptoms, since constipation, for some, is also considered synonymous with nasal congestion.

In the first question, “Frequency of bowel movements”, the consensus version was “*Frequência das evacuações*”, due to the probable misunderstanding arising from the literal translation “*frequência dos movimentos intestinais*”. On the first score line, “1-2 times per 1-2 days”, the consensus was “*1 ou 2 vezes a cada 1 a 2 dias*”. The option for “*ou*” instead of “*a*” was due to the more important aspect of 1 to 2 days in relation to how many bowel movements were within this period. The remaining lines received a literal translation.

The second question: “Difficulty: painful evacuation effort” received the consensual translation “*esforço evacuatório desconfortável ou doloroso*”. The suggested option, “*esforço doloroso para evacuar*”, was disregarded, as well as the literal translation, emphasizing the binomial “evacuation effort” and not the word “difficulty”, favoring the concept of the symptom, frequent in intestinal constipated patients. The scoring lines have been literally translated, with “*geralmente*” being the accepted translation for “usually”.

In the third question, “Completeness: feeling incomplete evacuation”, the consensus version was “*sensação de evacuação incompleta*”, considered simple and adequately expressing the question concept, and greater objectivity of the binomial “incomplete evacuation”. The option “*esvaziamento retal: sensação de evacuação incompleta*” was disregarded due to the presumed difficulty in understanding the term “rectal”. The translation “*esvaziamento: sensação de evacuação incompleta*” or “*sensação de esvaziamento incompleto*” was also disregarded when compared to the option chosen, due to its objectivity. The scoring lines are identical to the second question.

The following question, “Pain: abdominal pain”, was consensually translated as “*dor abdominal ao evacuar*”, relating the symptom with defecation. The scoring lines are identical to the second question.

The fifth question: “Time: minutes in lavatory per attempt” was translated as “*tempo no banheiro para evacuar (em minutos*)”, emphasizing the binomial “time” and “evacuation”. The options “*tempo em minutos no banheiro para evacuar*”, “*tempo: minutos gastos no banheiro por tentativa de evacuação*” and “*tempo: tempo (minutos) gasto no banheiro em cada tentativa para evacuar*” were considered longer and more difficult to understand than the accepted option. The scoring lines were adapted as follows: from “5-10” to “*5 a 10 minutos*”, maintaining the same rationale for the remaining scoring lines.

The question “Assistance: type of assistance” received the consensus version “*uso de laxantes, enemas ou auxílio com as mãos*”. The title of this question containing all the possible options made it clearer. Considering the study population, the literal translation “*assistência: tipo de assistência*” as well as the others: “*manobras ou medidas para ajudar a evacuação*”, “*ajuda: tipo de ajuda para evacuar*” and “*recurso: ajuda para evacuar*” were considered less objective and allowed misunderstandings. The first scoring line “without assistance” has been translated as “*sem ajuda*”. The line “stimulative laxatives” was adapted to “*uso de laxantes*”, and the following line, “digital assistance or enema” to “*uso de enemas ou ajuda com as mãos*”. The word “*enemas*” was used in plural to differentiate from the occasional use commonly requested as preparation for some complementary exams, such as rectosigmoidoscopy, for example.

In the seventh question: “Failure: unsuccessful attempts for evacuation per 24 hours”, the idea of the question concept was preferred: “*tentativas malsucedidas (fracassadas) para evacuar*”, associating the binomial “unsuccessful attempts” with evacuation. According to the specialists, other options such as “*fracasso: tentativas malsucedidas de evacuação em 24 horas*” and “*fracasso: tentativas fracassadas de evacuação por 24 horas”* or “*tentativas frustradas de evacuação por 24 horas*” diverged from the focus, emphasizing the word “failure”. When starting with “attempts”, the title became more objective and linked to the defecating act. Again, in the scoring lines, “1-3”, as an example, has been adapted to “*1 a 3 vezes por dia*”, considered easier to understand. The remaining lines follow the same pattern.

The eighth question, “History: duration of constipation (yr)*”* was conceptually adapted to “*duração da constipação (em anos)”,* associating intestinal constipation with its duration in years. Scoring lines have been adjusted from, for example, “1-5” to “*1 a 5 anos*”, improving this time range comprehension.

Cultural adaptation was the first step in using the definitive version for the Portuguese language spoken in Brazil. The global content validity index, meaning the understanding level of the instrument by the interviewee, was significantly higher than 80%^6^, considered excellent to any kind of measurement instrument. The Constipation Assessment Scale^25^, which chronologically preceded the Constipation Scoring System^1^, obtained a content validation of 75%, considered only moderate. The translation and validation into Portuguese of the Fecal Incontinence Quality of Life^35^ reinforces the importance of cultural adaptation, which is not always carried out during validation studies^6^, presenting items comprehension by respondents of only 40 and 0%, for example, as in the items “*2g*” and “*3l*”, respectively, as described above.

The questions with the lowest CVI were numbers 3 and 7, “*Sensação de evacuação incompleta*” and “*Tentativas malsucedidas (fracassadas) para evacuar*”, respectively. In question three, despite being a very common symptom - the average score was three, ranging from zero to four - the word “incomplete” could have generated some initial misunderstanding. In general, patients whose “I did not understand” option was marked had less than four years of schooling. Question seven is formed by a less prevalent symptom, arising difficulty in comprehension of the related concept. Among the eight questions that comprise the instrument, it was the least scored.

Considering the validation itself, reliability assessment is the one that simulates real life aspects, such as in clinical practice, in patient’s evaluation and follow-up, as well as in scientific research, as in multicenter studies.

Interobserver and intraobserver evaluation ratified the applicability of the instrument, regardless of temporal aspects and minimizing perception bias, when subjective aspects of the interviewer’s interpretation can interfere with the measurement^15^.

The concept idea of each question and the simplicity of the scoring lines (never, rarely, sometimes, usually, and always, for example), facilitate the reproducibility of the *Constipation Scoring System/ Índice de Gravidade da Constipação Intestinal*.

The ICC quantifies the degree of reproducibility of an instrument: the *Índice de Gravidade da Constipação Intestinal* presented excellent global ICC, both in the interobserver and intraobserver analysis, with statistical significance. This data becomes even more important when compared to other studies. Validation of the Constipation Assessment Scale^25^ obtained a moderate Cohen’s kappa coefficient (0.714). Validation of a 28-item dental questionnaire applied to 130 individuals obtained moderate overall consistency (0.7)^24^. The PAC-SYM^13^ and PAC-SYM for the use of opioids^31^ also obtained moderate reliability, respectively ICC equal to 0.75 and Cronbach’s alpha test greater than 0.7. On the other hand, the KESS score^18^, using the Constipation Scoring System^1^ as one of its validity instruments and also based on complementary tests for the diagnosis of intestinal chronic constipation, obtained an excellent Pearson correlation coefficient (0.9).

In the intraobserver evaluation, there was the only question with moderate ICC. In this form of analysis, the patient responds to the researcher at different time points - in this study, seven days. Question 7 refers to “*Tentativas malsucedidas (fracassadas) para evacuar*”. As also seen in the other stages of the Constipation Scoring System’s validation with patients, this is the one that received the lowest scores. The reduced prevalence - which can generate difficulty in assimilating the question concept - associated with a one-week interval (bias of the information recall period)^30^ may be related to the moderate ICC. Measurement instruments whose items have a moderate ICC (values greater than 0.5) with statistical significance are considered valid for use^20^, a common situation for questionnaires with less than ten items^24^.

Graphically, the Bland-Altman analysis (Figure 6) demonstrated the significant agreement between both assessments, showing only two points of disagreement beyond the regions of acceptance of the graphic in the interobserver analysis, considered largely satisfactory^20^ ^,^ ^24^.

As the measurement instrument exists to quantify some condition, in the absence of symptoms, as in the case of volunteers during divergent validation, the reliable tool must indicate this normality, withstanding a score closest to the minimum. In a score that can range from zero to 30, the average global score was much lower than the 15-point limit related to the diagnosis of constipation. Similarly, the KESS score^18^, in turn, obtained an average of 20 points for patients and only two points for volunteers.

Regarding patients who had a good response to clinical treatment, the instrument is expected to clearly present the diagnosis and its severity as well as to demonstrate a significant scoring decrease after successful treatment.

In this analysis group, the tool “*Índice de Gravidade da Constipação Intestinal*” significantly indicated the diagnosis, its severity, and its decrease after the treatment. Similarly, the PAC-SYM for the use of opioids obtained good responsiveness^31^.

Precisely, it indicated the invariability of question number 8, “*Duração da constipação (em anos)*”, which is not affected by treatment, due to its temporal concept of symptoms duration.

In the convergent validation of refractory patients, the average score was greater than 20 points, in a tool that varies from zero to 30, indicating the severity in patients with established clinical treatment. Likewise, the Japanese translation and validation of the PAC-SYM Quality of Life^33^ varied accordingly to constipation severity when compared to the Constipation Scoring System^9^.

In regarding female gender as a risk factor for intestinal chronic constipation, considering a group refractory to clinical treatment, it can be inferred that the female-male ratio becomes more significant. In this group, the proportion of women was higher than any other patient group.

The application of the *Índice de Gravidade da Constipação Intestinal* to clinically refractory patients showed higher scores when compared to those participating in the cultural adaptation, validation - reliability and convergent validation group of good response to clinical treatment (pre-treatment).

By comparing the group of refractory patients with the volunteers (divergent validation) and those in the convergent validation group with good response to clinical treatment, we clearly verify the *Índice de Gravidade da Constipação Intestinal’s* sensitivity and its responsiveness.

In general, all measurement instruments have limitations and an interesting dilemma: the more complete they are, the more reliable and objective the information will be. However, they will be more complex and of a longer completion, creating different difficulties and biases, at risk of being relegated to disuse. The current tool linked a reduced number of questions and agility in its completion to a high accuracy.

The Constipation Scoring System and *Índice de Gravidade da Constipação Intestinal* overlap intervals in the score lines of questions 5, 7 and 8. In question 5, for instance, where the second score line (*de 5 a 10 minutos*) and the third line (*de 10 a 20 minutos*) overlap between “*10 minutes*” from the second line, scoring 1 point, and “*10 minutes*” from the third line, scoring 2 points, the definitive version stated the maintenance of this interval pattern to preserve the essence of the original instrument. Also, as it was considered simpler for the population to be interviewed and due to the aspect of the concept, the idea of the time indicated by the interviewee being subjective and approximate, not being objective and timed in everyday life.

The sample amount calculations were not carried out with this purpose: agreement, similarity and compatibility between measurement instruments and not aiming to obtain incidence, prevalence, or risks. Even so, the number of respondents in all stages was greater than 30, a sample considered significant in instrument validation studies^31^.

This severity score was created for the diagnosis and follow-up of intestinal chronic constipation, which has a high national prevalence, without being directed towards any specific etiology, as occurred with most of the scores that emerged after its creation^2^ ^,^ ^11^ ^,^ ^19^ ^,^ ^21^ ^,^ ^22^ ^,^ ^34^.

The ideal instrument should keep the pace of the evolution and changes that occur in the population for which it was intended, being equally dynamic. Future comparative studies with other tools, such as those of specific etiologies^2^ ^,^ ^11^ ^,^ ^19^ ^,^ ^21^ ^,^ ^22^ ^,^ ^34^, may bring new information and perspectives on the evaluation, treatment, and follow-up of patients with intestinal chronic constipation.

## CONCLUSIONS

Translated into Portuguese and validated for use in Brazil, the *Índice de Gravidade da Constipação Intestinal* is a reliable instrument as a score of severity of intestinal chronic constipation.
